# Abstract of Proceedings of the Buffalo Medical Association

**Published:** 1867-11

**Authors:** T. M. Johnson

**Affiliations:** Sec’y.


ART. V.—*Abstract of Proceedings of the Buffalo Medical Association.*

TUESDAY EVENING, October 1st, 1867.

The meeting was called to order at the usual hour by the President. Members present—Drs. Eastman, Potter, Daggett, Smith, Kamerling, Boardman, Congar, Strong, Wetmore and Johnson.

The minutes of the last meeting were read and approved.

DR. BOARDMAN, Chairman of the Committee on Constitution and By-Laws, presented the report of the committee and pointed out the proposed changes in the By-Laws. He also reported a Fee Bill for the consideration of the Association.

Considerable informal discussion was had upon the subject of the above report.

The President informed the meeting that final action would be had upon the Constitution and By-Laws at the next regular meeting.

DR. BOARDMAN said he would like to inquire if any of the members had seen any unusual sequele of scarlatina? He had seen an unusual tendency to pain in the lower extremities, with swelling and slight redness coming on after the rash and fever had subsided, and lasting three or four days.

DR. EASTMAN said that he had seen several cases of scarlatina within the month, but had not seen any of the peculiarities mentioned by Dr. Boardman.

DR. STRONG had seen no such cases this year, but had in former years.

No especial disease was reported as prevailing.

Dr. Henry Nichell was elected to read an essay at the regular December meeting.

Adjourned. T. M. Johnson, M. D., Sec'y.

